# Understanding Needs for Chronic Musculoskeletal Pain Management in a Northern Dene and Métis Community: A Community Based Needs Assessment

**DOI:** 10.1080/24740527.2024.2412560

**Published:** 2024-12-06

**Authors:** Tayah Zhang, Brenna Bath, Veronica McKinney, Jaris Swidrovich, Rachel Johnson, Heather Foulds, Nadia Makar, Melanie Montgrand, Stacey Lovo

**Affiliations:** a College of Medicine, University of Saskatchewan, Saskatoon, Saskatchewan, Canada; b School of Rehabilitation Science, University of Saskatchewan, Saskatoon, Saskatchewan, Canada; c Northern Medical Services, University of Saskatchewan, Saskatoon, Saskatchewan, Canada; d Leslie Dan Faculty of Pharmacy, University of Toronto, Toronto, Ontario, Canada; e Angelique Canada Health Center, Pelican Narrows, Saskatchewan, Canada; f College of Kinesiology, University of Saskatchewan, Saskatoon, Saskatchewan, Canada; g Saskatchewan Health Authority, Primary Health, Northwest; h College of Education, University of Saskatchewan, Saskatoon, Saskatchewan, Canada

**Keywords:** Chronic musculoskeletal pain, Indigenous perspectives, Métis, Dene, needs assessment, pain management

## Abstract

**Background:**

Chronic musculoskeletal (MSK) pain disproportionately affects Indigenous Peoples, and rural/remote communities face significant barriers in accessing care. La Loche, a Dene/Métis community in northern Saskatchewan, has limited access to specialized chronic pain management services and specialized health providers.

**Aims:**

The aim of this needs assessment was to gain insight into the community’s priorities, strengths, and concerns regarding chronic MSK pain management. Community engagement and relationship building were essential to ensure that cultural protocols were respected and community worldviews were accurately represented.

**Methods:**

A community-directed needs assessment was conducted in collaboration with local health care providers and community members. To ensure appropriate representation of community-led priorities, reflexive thematic analysis was utilized and rooted within interpretive description and informed by community-based participatory research and Two-Eyed Seeing. Open discussions were conducted in person, over the phone, or via Zoom in a semistructured format. Thirteen individuals were interviewed (eight community members, five health care professionals).

**Results:**

Interviews conducted with community members and health care providers were analyzed separately. Both yielded the same four major overarching themes: (1) impact of pain on daily living, (2) barriers limiting access to care and the understanding of pain between health care provider and patient, (3) systemic oppression and negative experiences with health care, and (4) strength-based solutions.

**Conclusions:**

Five recommendations were developed to promote culturally safe and patient-centered environments for chronic MSK pain communication and future care delivery: (1) person-centered and community-directed care, (2) clinic model and staffing requirements, (3) practitioner education and awareness, (4) community education and awareness, and (5) community resources.

## Résumé

**Contexte :** La douleur musculosquelettique chronique affecte de manière disproportionnée les peuples autochtones. De plus, les communautés rurales éloignées font face à des obstacles importants pour accéder aux soins. La Loche, une communauté dénée/métisse du Nord de la Saskatchewan, n'a qu'un accès limité aux services spécialisés de prise en charge de la douleur chronique et aux prestataires de soins spécialisés.

**Objectifs :** L'objectif de cette évaluation des besoins était de mieux comprendre les priorités, les forces et les préoccupations de la communauté en ce qui concerne la prise en charge de la douleur musculosquelettique chronique. L'engagement de la communauté et l'établissement de relations étaient essentiels pour s'assurer que les protocoles culturels étaient respectés et que les visions du monde de la communauté étaient représentées de manière fidèle.

**Méthodes :** Une évaluation des besoins orientée vers la communauté a été menée en collaboration avec les prestataires de soins de santé locaux et les membres de la communauté. Afin d’assurer une représentation appropriée des priorités communautaires, une analyse thématique réflexive a été réalisée, ancrée dans la description interprétative et éclairée par la recherche participative communautaire, ainsi que par l’approche de « vision à deux yeux ». Des discussions ouvertes ont été menées en personne, par téléphone ou via Zoom dans un format semi-structuré. Treize personnes ont été interrogées (huit membres de la communauté, cinq professionnels de la santé).

**Résultats :** Les entretiens menés avec les membres de la communauté et les prestataires de soins ont été analysés séparément. Les deux types d'entretiens ont permis de dégager les quatre mêmes thèmes principaux : (1) les effets de la douleur sur la vie quotidienne, (2) les obstacles limitant l'accès aux soins et la compréhension mutuelle entre le prestataire de soins et le patient en ce qui a trait à la douleur, (3) l'oppression systémique et les expériences négatives avec les soins de santé, et (4) les solutions fondées sur les forces.

**Conclusions :** Cinq recommandations ont été élaborées pour promouvoir des environnements culturellement sûrs et centrés sur le patient en ce qui concerne la communication et la prestation des services dans l’avenir : (1) des soins centrés sur la personne et dirigés par la communauté; (2) le modèle de clinique et les exigences en matière de personnel; (3) la formation et la sensibilisation des praticiens; (4) la formation et la sensibilisation de la communauté; et (5) les ressources communautaires.

## Background/Introduction

Musculoskeletal (MSK) conditions are result of impairments of the bone, muscle, joints, ligaments, or tendons.^1^ MSK disorders are among the most prevalent health conditions, affecting 1.71 billion people worldwide.^2,3^ Based on disability-adjusted life-years calculations in 2017, MSK disorders ranked fifth highest in terms of overall disease burden.^2,4^ MSK conditions are often associated with pain, limitations on mobility, and reduced quality of life.^1,5^ The International Association for the Study of Pain describes pain is as “an unpleasant sensory and emotional experience associated with, or resembling that associated with, actual or potential tissue damage.”^6^ Pain experienced with MSK conditions can be acute (fractures) or chronic (low back pain).^1^ The International Association for the Study of Pain defines chronic pain as pain that persists for more than 3 months.^7^ Chronic MSK pain negatively impacts daily function, sleep quality, employment, social participation, life role fulfillments, and mental health.^5,8^ The number of individuals living with chronic MSK pain is increasing.^4^

**Figure 1.**
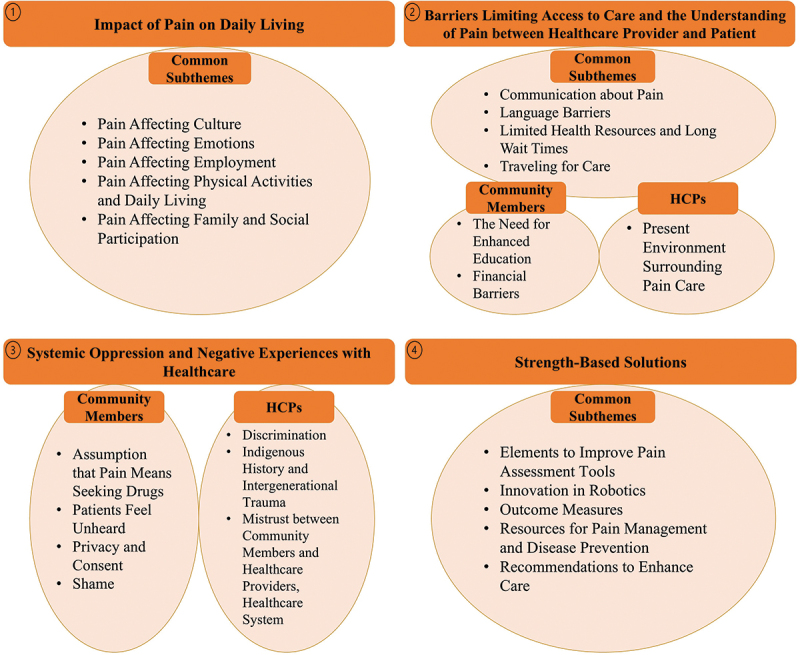
Outline of La Loche community member and health care provider themes. The four major themes with their subthemes are outlined. Common subthemes are subthemes that are shared between community members and HCPs. Subthemes that are unique to each group are listed below their respective headings.

MSK disorders reported by Canadians in 2017 included low back pain (15.5%), osteoarthritis (7.5%), neck pain (3.5%), gout (1.4%), rheumatoid arthritis (0.35%), and other MSK disorders (6.9%).^9^ Chronic MSK pain involves social, psychological, financial, and environmental factors that are all unique to each person’s lived experiences.^10,11^ Due to the complexity of chronic MSK pain, comprehensive pain assessments by a multidisciplinary health care team and a biopsychosocial model of care is considered best practice.^10,11^ Chronic MSK pain management guidelines encourage minimal medication use by increasing preventative education and nonpharmacological approaches.^5^ A holistic approach is recommended in the context of the patient’s lived experiences, current lifestyle, preference, culture, and mental health.^5,10^

Indigenous Peoples in Canada (Inuit, Métis, and First Nations) are disproportionally impacted by higher rates of pain and pain related disability than non-Indigenous people.^12^ Primary reasons for seeking health care include the management of chronic pain.^12–14^ Information on chronic MSK pain among Indigenous Peoples in Canada is sparse. The Canadian Pain Task Force^11^ reported that Indigenous Peoples experience the highest prevalence of chronic pain in Canada compared to other Canadian populations and MSK conditions are commonly the reason for those chronic pains. Rates of low back pain, rheumatoid arthritis, osteoarthritis, and other MSK conditions are higher among Indigenous Peoples in Canada when compared to non-Indigenous populations.^15,16^ Indigenous Peoples within rural locations in Canada have a 30% greater chance of experiencing chronic low back pain than non-Indigenous individuals.^17^ In the face of the significant prevalence of chronic MSK pain, Indigenous Peoples continue to persevere despite the persistent disparities they face in access to care for pain management options.

Regardless of established MSK pain management guides, many Indigenous Peoples still face inequitable pain management such as inappropriate opioid prescriptions, lack of specialist referrals, lack of access to care, and racism within the health care system.^13,18–20^ Indigenous Peoples are more likely to be dispensed an opioid than non-Indigenous individuals and they are more likely to experience an opioid-related overdose than non-Indigenous people.^20,21^ Factors that may affect the high level of opioid prescription within Indigenous populations include the limited access to health resources and the challenges with continuity of care.^22^ Webster^22^ reported that northern Indigenous communities in Ontario are often located in remote regions where “fly-in” physicians visit at scheduled times. Within these communities, many Indigenous Peoples who are dependent on opioids trace their dependency to the “fly-in” physicians who work for Health Canada on short-term contracts.^22^ The lack of continuous care leads to weak foundational relationships between physician and patient, and the lack of alternative resources to pain management may generate a trend of extensive opioid prescribing.^22^ However, to avoid exacerbating health inequities and the impacts of pain, efforts should also be made to avoid unduly refusing to prescribe opioids when indicated to avoid unintended harms to those who use opioids for pain management.^23,24^

Pain is interrelated with discrimination, trauma, social marginalization, invalidation of the pain experience, mental health, and health care inequities.^25^ Indigenous Peoples’ pain is often dismissed by health providers due to biases and racist treatment in the health system, including the assumption of drug-seeking behaviors.^13^ Racialized health care experiences of Indigenous Peoples are rooted throughout Canadian history (and experienced presently), which must be reviewed in the context of the nation’s colonial actions. Indigenous Peoples have fought for their identity and culture throughout oppressive, genocidal policies and practices such as residential schools, the Indian Act, the 60s scoop, and the sterilization of Indigenous women.^26^ Indigenous Peoples across Canada are continually impacted by the long-lasting harm of the historical traumas.^26^

Mathur and colleagues^27^ suggested that pain disparities faced by racialized groups are direct results of injustices at the cultural, structural, and interpersonal levels. Racialized oppression shapes the lived pain experiences of patients and increases the risk of poor pain outcomes.^27^ Mathur and colleagues^27^ explained cultural injustice as inequalities that are built into language, society, values, and worldviews. Structural injustices involve inequalities built into governments and health care systems whose policies and practices exploit Indigenous Peoples’ right to vote, receive education, receive equal opportunities, obtain housing, secure employment, and access equitable health care.^27^ These factors impact an individual’s pain outcomes and lead to pain disparities. Explicit biases, implicit biases, and discrimination are considered interpersonal injustice, which can amplify the degree of pain physically, emotionally, psychologically, and spiritually.^27^ Implicit and explicit biases toward Indigenous Peoples in Canada are well documented and discussed on social media and in provincial reports in British Columbia, Alberta, and Ontario.^19,28,29^ Implicit biases lead to discrimination experienced by Indigenous patients such as “abusive interactions, denial of service, inappropriate pain management, ignoring or shunning.”^29^ In a 2020 government-commissioned report of anti-Indigenous racism in British Columbia, only 16% of the total number of Indigenous participants who responded to the survey (*n* = 2780) had not experienced discrimination or stereotyping when accessing health care.^29^ More than one-third of the total surveyed health care providers (HCPs; *n* = 5440) reported witnessing discriminatory acts toward Indigenous patients and families.^29^ Persistent negative experiences of injustices, racism, discrimination, and poor health care will compromise an individual’s views on health care, which can lead to avoidance of care and lack of preventative care and education.^30,31^

Although it is important to understand the literature surrounding MSK pain and pain disparities experienced by Indigenous Peoples, it is also vital to not presume that each Indigenous community experiences similar challenges, because every community possesses unique strengths, cultures, histories, identities, and access to social determinants of health.^19,32^ A community needs assessment is a beneficial way to understand a community’s strengths and concerns around MSK pain management. It can lead to community-identified health outcomes and an increase in the awareness of the needs, views, and preferences of community members to ensure that future interventions are tailored to the community.^33^ To ensure meaningful relationships with community members, continuous community engagement that prioritizes the community voices is required.^34^

A needs assessment helps identify ways in which current policies, services, barriers to access, and health resources within the community can be improved to benefit residents and their pain experiences. Partnership with community members prioritizes their involvement in the research process to ensure relevant and practical future interventions. The aim of this needs assessment was to uplift community members’ and HCPs’ voices to understand the community’s current efforts in chronic MSK pain management and to capture community-identified recommendations that would be suitable for the community in future virtual health care interventions for MSK pain management. This is phase 1 of a two-phase project occurring in two different Indigenous communities in northern Saskatchewan.

## Methods

### Setting

La Loche is a village located in northern Saskatchewan with a population of 2514 in 2021.^35^ In the 2021 census, the community of La Loche comprised 94.2% Indigenous residents, of whom 35.7% are Métis and 56.3% are First Nations (specifically Dene).^35^ In the same census, 51.7% of community members mostly speak Dene at home and 36.5% of members speak English.^35^ The Saskatchewan Health Authority and Northern Medical Services oversee health care services offered within La Loche and surrounding communities (estimated to be around 4000 individuals).^36^ Due to the community’s remote nature and distance from urban centers, access to specialized chronic MSK pain management services is reduced.

We worked in partnership with the community members and local HCPs to understand the strengths, concerns, and priorities of La Loche in terms of chronic MSK pain management. Community members’ voices provided insights on culture, language, and strengths and needs of the community and ensured that worldviews and protocols were respected.

### Framework

Interpretive description with analytic procedures of thematic analysis, informed by community-based participatory research and Two-Eyed Seeing, was utilized throughout this needs assessment to ensure that the project was culturally relevant and community-led.^35–40^ Interpretive description is based in an interpretive paradigm that recognizes that realities are socially constructed with elements of the human experience.^41^ It is often used by health professions when the aims of the project are to understand subjective experience, to identify themes and patterns of a population, to apply the knowledge in real-world applications, and to inform better clinical practice.^41^ Interpretive description follows the generic qualitative research process where data are collected in the form of interviews, field notes/researcher journaling, and observations followed by analysis of data in the form of coding.^41^ Thematic analysis was used to identify patterns and themes from a “bottom-up” manner where the codes were generated from raw data and themes from codes.^42^ Ultimately, major themes were constructed to help the researchers understand the phenomenon from the participants’ subjective experiences.^41^ This methodology was in keeping with our goal of having actionable outcomes to inform phase 2 of the project to co-develop a virtual health care intervention for chronic MSK pain management within the community.^41^

Community-based participatory research ensures a collaborative partnership where researchers and community members will be treated as equals.^37,40^ It allows for community members to lead and identify community strengths, needs, and desired health outcomes.^37,40^ Two-Eyed Seeing is a research guide for Indigenous inquiries that combines knowledge of both Western and Indigenous worldviews.^43^ Two-Eyed Seeing was the overarching framework, allowing the outcome of this project to integrate the strengths of Indigenous and Western ways of knowing while prioritizing Indigenous knowledge and experiences. This project facilitated a collaborative discussion *with* Dene/Métis community members about chronic MSK pain and pain management in hopes of improving future health care experiences and health outcomes.

The lead author of the article is a graduate student and immigrant to Canada studying health equity and lived pain experiences of Indigenous Peoples in northern Saskatchewan. The senior author is a fourth-generation settler ally who works in partnership with First Nations and Métis communities in Saskatchewan to enhance health care experiences. Coauthors include a Cree and Métis physician scholar, Métis and First Nations scholars, a member of the community of La Loche, and scholars in the field of physiotherapy and virtual health care. The authors were careful to be reflective in analysis and undertook repeated review to maximize their diverse backgrounds and expertise to reduce potential bias. Additionally, to ensure appropriate interpretation of experiences, community members were involved through all stages to ensure that Indigenous worldviews were prioritized.

### Ethics

Ethics approval for this project was received through the University of Saskatchewan Ethics Board (Beh ID 3016) in March 2022, which also included a separate review within the community of La Loche. A Saskatchewan Health Authority operational approval was received in July 2022. All members of the research team completed the *Tri-Council Policy Statement: Ethical Conduct for Research Involving Humans*, *2nd Edition*^44^ tutorial for research involving participants.

### Community engagement procedure

The research team’s initial focus was on fostering a meaningful relationship with the community of La Loche. Indigenous scholar V.M. has a longstanding relationship of over 10 years with the community of La Loche, local governance leadership, and the health facility. Other team members (S.L., B.B.) had worked with local HCPs and a Dene language keeper for 3 years in previous community-directed projects. In working with the community mayor and the local health clinic, a local Dene-speaking community research assistant (CRA) was hired. The CRA played a vital role in ensuring that community protocols and culture were respected and provided education on the importance of language in promoting health, wellness, and trust. We were committed to ensuring that traditions and protocols of La Loche were followed; therefore, we sought guidance from Elders. To ensure that the community members felt safe and respected, the CRA played a critical role as a translator for those who wished to participate in Dene. Members of the research team visited the community in June 2022 for a day of community engagement and relationship building. During their visit, they toured the community, got to know some of the community members, and sought guidance from the Dene language keeper and Elder.

### Recruitment and data collection procedure

Recruitment posters were shared to the community over Facebook, following the local CRA’s recommendations. Posters were also displayed in the health center and the mayor’s office. Individuals 18 years of age or older, from La Loche or surrounding communities, and experiencing chronic MSK pain (pain that has persisted for 3 months or more) were invited to share their experiences. Participants were provided honoraria, and Elders were offered gifts, tobacco, or jam in addition to honoraria for their knowledge sharing. In addition, HCPs who work with individuals living with chronic MSK pain were invited to participate and share their perspectives. Health care provider recruitment also occurred via posters in the health center and through word of mouth. Additionally, to increase awareness, nurses were informed of recruitment through other staff at the health care center and physicians were informed through the Northern Medical Services for participation; however, there were no volunteers for participation from these two professions. Participants gave informed written consent with in-person interviews, and participants who connected virtually gave informed verbal consent.

Interviews began in August 2022. A second community visit occurred in September 2022 for further relationship building and to conduct semistructured interviews. Indigenous scholars on our team (V.M. and J.S.) provided guidance in the creation and further revisions of the semistructured interview guide (Appendix A and Appendix B). Virtual semistructured interviews were also conducted using Zoom^45^ or a phone call. Interviews were led by the first author and facilitated by the CRA in Dene. The CRA acted as a translator in three interviews with community members. The interviews were audio recorded with the participants’ consent and transcribed at the Canadian Hub for Applied and Social Research (University of Saskatchewan). All participants were given the opportunity to review and revise their transcripts to ensure that all voices were accurately represented. A total of 13 individuals were interviewed: eight community members and five HCPs. Of the five HCPs interviewed, two identified as being Indigenous. All community members who were interviewed completed a pre-interview demographic survey. All interviews were one-on-one except for two community members who preferred to do the interview together because they were family and their MSK pain experiences were closely intertwined. Of the eight community members, four identified as male and four identified as females. The average age was 48 years old with a median of 54 years of age. Five community members shared that they smoke, two indicated they have never smoked, and one participant used to smoke but no longer smokes. All eight community members expressed that they are experiencing current episodes of chronic MSK pain. Community members expressed an average of 10.9 years of long-standing MSK pain. In terms of comorbidities, 8/8 experience headaches, 6/8 experience lung/breathing problems, 3/8 experience heart problems, 6/8 experience stomach/digestive problems, and one participant is a breast cancer survivor. No other major comorbidities were disclosed.

### Analysis

Transcripts were thematically analyzed using NVivo, a qualitative analysis software.^46^ Analysis began in November 2022. To enable the identification of potentially differing themes, the HCPs’ transcripts were analyzed separately from those of community members. The process involved initial familiarization of the transcripts, followed by further readings focused on identification of recurring patterns and ideas within each transcript. Patterns and ideas across transcripts were then identified to develop the preliminary codes that were subsequently categorized into emerging themes. A hierarchy was established, with major themes emerging from subthemes and sub-subthemes. The researcher (T.Z.) and principal investigator (S.L.) engaged in regular discussions to ensure reliability throughout the analysis process. Indigenous scholars (V.M. and J.S.) were included in the transcript analysis to ensure Two-Eyed Seeing procedures. Discussions among T.Z., S.L., V.M., and J.S. facilitated the identification of redundant codes and restructuring, as well as organization of codes into detailed subthemes. Additionally, Indigenous scholar H.F. further refined the organization of the themes and ensured that the creative process for the figure incorporated an Indigenous perspective.

## Results

Community members of La Loche reported a range of MSK conditions, including low back pain, osteoarthritis, neck pain, and rheumatoid arthritis, which are all among the most common MSK conditions. All eight community members reported experiencing osteoarthritis. Though one individual solely lived with osteoarthritis, the other seven experienced multiple MSK conditions: four reported osteoarthritis with low back pain, two reported osteoarthritis with low back pain and neck pain, and one reported osteoarthritis with rheumatoid arthritis and neck pain.

Qualitative findings through separate discussions with community members and HCPs yielded four major themes in common between community members and HCPs: (1) impact of pain on daily living, (2) barriers limiting access to care and the understanding of pain between health care provider and patient, (3) systemic oppression and negative experiences with health care, and (4) strength-based solutions. Each major theme has its own subthemes and sub-subthemes, which differed slightly between conversations with community members and HCPs. Figure 1 outlines the themes. Appendixes C and D provide additional representative quotes from community members and HCPs, respectively, that were not included in the main text.

### Major theme 1: Impact of pain on daily living

Community members shared how MSK pain can affect multiple aspects of their livelihood, from impacting culture to negatively affecting emotions, employment, family relations, social participation, and directly limiting physical activities and function. The subthemes were as follows: (1) pain affecting culture, (2) pain affecting emotions, (3) pain affecting employment, (4) pain affecting physical activities and daily living, and (5) pain affecting family and social participation. HCPs recognized the impacts that MSK pain could have on community members’ daily lives across the five areas identified by community members.

#### Pain affecting culture

Community members expressed that MSK pain has limited their ability to practice their own culture and traditional way of living, including participation in ceremony, hunting, and fishing. One participant shared, “There’s these sweat lodges that we do, and I can no longer participate because of the pain that I feel with the heat. I can no longer go to traditional dances anymore. I can’t participate in traditional community activities. I can’t do a lot of things” (C5). Another participant expressed, “I probably couldn’t dance Powwow and I can’t even kneel down if my knee is sore. So, it’s hard for me to even get into a smudge or anything like that.” (C2)

Health care providers expressed that pain has limited patients’ ability to practice their culture and traditional way of living, including picking berries, hunting, and participation in spiritual activities. One HCP shared, “Participating in cultural or spiritual activities is affected because these individuals are not really going out into the community” (H5). Another HCP noted that social activities may have cultural significance, but they acknowledged a lack of understanding of what qualifies as a cultural practice.

#### Pain affecting emotions

Participants expressed feeling exhausted and frustrated living with constant pain. One participant expressed, “I get real bad anxiety and I feel like I can’t do stuff right now. I’m just really depressed. … It has made me not want to be around people so much because I do get irritated” (C1).

Health care providers conveyed that chronic pain has profound effects on patients’ emotional well-being, resulting in limitations on mobility and restricted participation in the community. They highlighted that lack of social participation can lead to adverse emotional effects. They said, “These individuals have isolated themselves and it’s affecting them daily. Not leaving the home much, unless they need to. Their lives are definitely affected and they’re not really living because they’re trying to stay in their comfort zone most of the time” (H5).

#### Pain affecting employment

When speaking about pain experiences, participants expressed that pain has negatively impacted their employment status. For example, one participant shared, “For me, pain has just totally destroyed my life … it got to the point where I couldn’t work on pain meds. … And because of my pain, I’m unemployed. … My wage lost, everything” (C3).

Health care professionals shared that individuals living with pain may face challenges with employment and may need to make career adjustments to manage their pain effectively. They said, “Sometimes it’ll take a patient out of employment forever sometimes it might require a change in employment” (H1).

#### Pain affecting physical activities and daily living

Community members expressed challenges with sleeping, household and outdoor chores, walking, lifting, bending, and appetite. One participant stated, “It’s even hard sleeping at night. My legs have to be a certain way and, yeah, I can’t do too much. Or even eating and stuff like that” (C1).

Community members living with pain encounter barriers in their daily lives, including mobility challenges like walking. An HCP shared, “All of them are unable to do certain things in their lives that they would really like to be able to do. There are lots of conversations about them wanting to get back to doing this or this is causing me trouble now I can’t do this thing anymore” (H3).

#### Pain affecting family and social participation

Participants reported that their pain has influenced their family responsibilities and social participation. Specifically, they shared that pain has acted as a barrier when it comes to engagement in social environments. A participant reported, “Some days it’s tough and other days, it’s fine but I find myself that I don’t try to get involve in things cause I’m afraid to make it sore. … I find that I don’t even like to have company because you can’t really visit or nothing” (C2).

Health care providers shared that community members have communicated experiencing social isolation as a result of limited mobility due to chronic pain. One individual expressed, “It’s difficult for them. If you’re not going anywhere and you’re not seeing much of anybody, then you’re just basically keeping to yourself and your family” (H5).

### Major theme 2: Barriers limiting access to care and the understanding of pain between health care provider and patient

The second theme that emerged described factors that challenged communication about pain between HCPs and patients. The community members shared experiences regarding accessing health care for pain management. The subthemes included (1) communication about pain, (2) language barriers (3) limited health resources and long wait times, (4) traveling for care, (5) the need for enhanced education, and (6) financial barriers. HCPs had the same first four themes as per community members but did not discuss the need for enhanced education or financial barriers. The fifth subtheme for HCPs was present environment surrounding pain care.

#### Communication about pain

Participants revealed a preference for describing their pain verbally along with motions to indicate the location of the affected area. Some community members shared that they had never used a pain scale before during pain assessments with HCPs. Of those who had utilized a pain scale, the majority had only utilized the numerical pain rating scale and had utilized a visual skeleton to aid in communication. A participant said:

They’d give me a pain scale which said which level is where and all that stuff. They’d sit there with a human skeleton and whatever, they’d draw circles where your pain was and everything. That was Dr. [deidentified], they’d tell you, “Point it out on a pain scale.” … On the skeleton drawing you could say, “Well my left arm is sore. From yesterday till today the worst pain is seven.” (C3)

Health care providers shared their experience with communication about pain management in the community, noting that traditional pain scales may not always be effective and identified that patients may prefer to communicate about their pain experiences through storytelling. Providers noted a potential disconnect in the understanding of pain between themselves and community members. An HCP shared, “I’m just using mostly the visual analogue scale and with that one, sometimes I feel it doesn’t trend. Maybe it’s just because they haven’t heard it before, or maybe it’s because it’s not geared to that population. It doesn’t always seem to make sense right off the bat” (H3).

#### Language barrier

Community members shared experiencing challenges when communicating with HCPs who do not speak Dene. Two individuals participated within one interview and spoke about the significance of the Dene language in understanding the culture and social norms of the community. They said:

Like, the Dene language, our language, the majority of the language is all facial expressions. Like, one word could mean a lot with different expressions, facial expressions. If you’re gonna come and work in La Loche, maybe you should take some course on Aboriginal—La Loche people, just how the Dene people are. … If you come to a three-year-old in La Loche, you talk to them in English, they won’t understand what you’re saying. … But if you talk to them in Dene, the three-year-olds would talk … in their native language, where they learn English in school as their second language. (C3)

Health care providers noted that effective pain communication requires attention to language differences. Specifically, community members of La Loche often speak Dene as their first language, whereas many HCPs primarily speak English. These linguistic differences can present a significant barrier to effective pain management. One HCP did not initially perceive language as a barrier; however, they were able to identify that many Elders in community often speak Dene as their first language, which can pose as a challenge when communicating. This HCP expressed the importance of having auditory formats of Dene to facilitate communication. They shared:

We do utilize our community outreach education worker to help translate as much as possible, but I would definitely say out of all the communities within our northwest region that where we’re going to see most language barriers for sure is in La Loche. It’s interesting because when we have resources that are in the Dene language most individuals don’t know how to read the Dene language, they just speak it. So, any resource really needs to be quite visual with pictures or audible. We did get—I’ve seen resources in my previous position that were made in the Dene language, and no one could read them.

#### Limited health resources and long wait times

Participants expressed that the remote nature of La Loche leads to a lack of health services and physician availability, which in turn affect the wait times. One participant described, “When you phone in the morning, they open at nine, right? But at five to nine, it’s busy and at ten after, it’s all booked up. I find it very hard to get an appointment” (C2). Participants also shared that the physicians who worked within the community were locum physicians and expressed concerns with a lack of continuity of care.

Health care providers expressed concerns with the wait time and waitlists in La Loche. They also provided insights on resources that could benefit the community, such as cognitive-behavioral therapy for pain management and an increase in staffing levels. An HCP shared:

I think, too, there’s a long waitlist, and if you’re needing to see them more than once every two weeks because of their patient load, that could be difficult. We just don’t have the capacity to be serving the people of La Loche sometimes as much as the community may need especially in relation to really anything in health care. (H2)

Another HCP stated that an increase in capacity of the health care clinic may benefit the community members.

#### Traveling for care

The idea of traveling for care was viewed through different perspectives by community members and HCPs. Community members expressed a preference for seeking health care services in alternative communities and urban centers due to past negative experiences in the community. Community members shared, “Yeah, I usually go to Battleford to get—better treatment” (C8). Two participants during one interview said:

used to go see the doctor in Saskatoon that we see had been assigned but, if not, I would go to St. Paul’s or University. Even though I was a Native, I communicated good with the doctors. A lot of them were interested in my Native culture background and everything. And we’d sit there for an hour or two or whatever and the doctor would jump up “I’ve got more patients, I forgot!” Not here, you know? They just—you’re not even allowed to warm up the seat a little bit. You stand there and you leave. I mean, it’s not that bad but it feels that bad. You go into the doctor’s office, and you have to sit there, wait for 45 minutes, no music, nothing. (C3)

Health care providers highlighted the challenge of transportation as a barrier to accessing health care for community members in La Loche. Given its remote location, the health clinic is the only facility available within a certain radius, which requires patients to secure transportation to attend appointments. Additionally, due to the size of the town, community members located within La Loche may also require transportation to access the clinic. Furthermore, the range of services available at the local clinic is limited, necessitating that patients travel to urban centers like Prince Albert or Saskatoon for health care. An HCP shared:

It is a larger town so it also could be a barrier to sometimes getting to the clinic, maybe difficult if you don’t have a vehicle or access to a vehicle or access to a ride that could be difficult because we don’t have public transportation. [Additionally], I think specialist appointments, sometimes those are—I think it can really be hard to get patients to a specialist appointment in the south even if you have coverage, like you are Treaty or you are on social assistance. … What if you have a bunch of children that need to be cared for? What if you don’t have a partner, like, and then you need to go the city, which can be considered really big and scary for a lot of people. It’s very traumatic. (H2)

Health care providers highlighted that due to the remote location of La Loche, travel expenses may be a limiting factor for some community members in accessing care. An HCP shared their personal experiences regarding traveling for health care and the financial burden it can impose. They stated, “We have to travel down south six and a half to seven-hour drive to Saskatoon to see a specialist. Those of us who are paying on our own, it’s a lot of money, a lot of our time, hotels and meals” (H5).

#### The need for enhanced education

Community members emphasized the importance of clear and effective communication between patients and HCPs to ensure informed decision making. One participant shared:

When you go to a hospital here, you just tell them how you feel or what’s hurting you. They don’t even check you. They are say[ing], “Just say take Advil or Tylenol.” … I stopped going to the hospital, because they don’t do nothing. They tell me to take Tylenol. … They don’t wanna explain much (C8).

Participants identified a need for enhanced awareness of available health services in community and enhanced health literacy. A few community members were not aware of all the services and tools that are available at the local health center, like physiotherapy; the remote presence robot, which can enhance service access; and the opioid antagonist treatment (OAT) program. One participant shared feeling frustrated and stigmatized, explaining, “Because there’s no community awareness on the methadone program, so people see you and think you’re a hardcore drug addict” (C3).

Health care providers did not identify components specific for enhanced education needs.

#### Financial barriers

A participant articulated the financial challenges they face in accessing health care, noting that although they have health care coverage, taking time off work to see a physician is not financially feasible because they have to prioritize work over their health to make ends meet.

#### Present environment surrounding pain care

This theme was found only in HCP data. Health care providers highlighted potential challenges in pain management, including lack of continuity of care, extended wait times for appointments, and lack of access to care. One individual shared:

[MSK pain] can be anywhere in the body but probably the most common areas are our low back, shoulders, knees, those would probably be the top 3 areas … there’s really zero consistency with who patients see at a primary care physician level. … 9/10 the person they’re seeing on follow-up is different doctor than they saw last time and that definitely doesn’t lead to ideal management when it comes to medications, ordering diagnostics, or following up on those. … Some consistency at the primary care level in terms of care providers would be really helpful, but that’s a much bigger systemic problem. (H1)

### Major theme 3: Systemic oppression and negative experiences with health care

The third theme highlights the ongoing systemic oppression that Dene/Métis community members have faced within the health care system. The subthemes that arose from conversation with community members include (1) Assumption that pain means seeking drugs, (2) Patients feel unheard, (3) Privacy and consent, and (4) Shame. Health care providers identified three different subthemes, and these will be highlighted following description of the community member subthemes.

### Community member subthemes

#### Assumption that Pain Means Seeking Drugs

Participants shared that HCPs often had preconceived notions that they were drug-seeking individuals despite expressing their desire for their pain to be treated seriously and requesting additional assessments to address their pain. One participant said, “They make me feel like I’m going there for pills, which I’m not even a pill-popper at all. They just make me feel like I’m there to get drugs, but I’m not. I really want x-rays done and stuff. They don’t take it seriously” (C1).

Two participants shared during one interview that they must manage their pain to practice their culture and sustain their livelihood. They conveyed that their pain is severely debilitating, to the extent that it is mentally exhausting. Their primary goal is pain management; however, when they seek care at the local health clinic, they are faced with stigmatization and presumptions that they are seeking medication.

##### Patients feel unheard

Community members also emphasized the importance of recognizing that people express pain in unique and varied ways. They cautioned against making assumptions or judgments based on appearances. Two participants echoed similar experiences. They provided an example:

You know, when you go to see the doctor at the hospital for whatever reason, the doctor and the nurse themselves say, “Okay, this is wrong with you, that’s what’s wrong with you.” They don’t listen to the patient, they decide what is wrong with the patient. Like I said, they decide for you. … They don’t acknowledge you. … If only the doctors would listen to the people like us, maybe our lives would be different today. (C4)

##### Privacy and consent

Participants shared their concerns regarding privacy and informed consent particularly in relation to the OAT program. They reported feeling stigmatized, and two patients even reported experiencing a lack of privacy that impacted their social lives within community:

Like the pharmacy, we said, there’s no privacy. So, the methadone is given to us right in front of everybody. And the people there on street drugs get their methadone, they sell it. And then they come and harass us that they need people to buy it off us. (C3)

’Cause it’s not given to us discreetly. If [it was given] to us discreetly, nobody would know we even get those meds. … There’s a few times we had to call the police on people that were bothering us. (C4)

Participants also shared their experience with lack of informed consent. They said:

There’s one day where we went to the pharmacy, we got to the pharmacy and we walked in, there was a doctor and a couple other people from Saskatchewan Health Authority standing there. … We were forced to sign something that we did not agree to. And we were forced to sign it because we were walking out sick. The doctor forced us to do that. Because I was trying to read the fine print and everything, he kept trying to pull it away from me. So finally, I had a pen, steel pen, I hit him on the finger as hard as I could. I told him I’m a slow reader. I did tell him three times I’m a slow reader. (C3)

##### Shame

Community members conveyed that they experienced a sense of shame when accessing health care for pain management. They said: “How do they treat us at the pharmacy when we’re on methadone? It’s 40 below, it doesn’t matter if the wind is whistling, snow blowing, we’re standing outside. People are coming and going at the pharmacy but if you’re on methadone, you stay outside like a dog until you’re called in” (C3).

## Health care provider subthemes

Health care providers identified discrimination and systemic oppression within the health care system. They emphasized the impact of intergenerational trauma and current discrimination on the establishment of trust and rapport between HCPs and Indigenous patients. Subthemes identified included (1) discrimination, (2) Indigenous history and intergenerational trauma, and (3) mistrust between community members and health care providers, health care system.

### Discrimination

Health care providers noted the negative effects of stereotypical views that are often directed toward community members, such as the perception of individuals as “drug-seeking.” They said:

I feel like the [health system’s] notes that I’ve looked at feel like these people are just always seeking medication. Which makes sense, if you’re in pain all the time you should be seeking out ways to manage your pain. I don’t think that the [health care system is] accusing these people of being drug-seeking. I do feel there’s a bit of tension there. People are asking for help and the medical system isn’t able to provide it for whatever reason; whether there just isn’t another drug that would help; we’ve tried all the options and unfortunately there just isn’t something, or whether the revolving door practitioners that visit La Loche just breaks down that continuity of care. (H3)

### Indigenous history and intergenerational trauma

Health care providers shared their knowledge of impacts of colonization that can affect the experiences of chronic pain, including involvement of the church, forced sterilization and birth control, intergenerational trauma, and the mistrust of systemic institutions. One HCP said:

There’re also mothers who maybe have a history with substance abuse but perhaps they’re actually really working on themselves. And so, for their entire pregnancy [they] want to change their lives and … their children are still taken away from them when they deliver. And that is scarring … and you now have a population of people who believe that the health authorities is ripping families apart … inadvertently. (H2)

### Mistrust between community members and health care providers, health care system

Health care professionals recognized that the systemic oppression experienced by the community has led to hesitancy in trusting the health care system. They noted that establishing rapport with community members can take time, but it is essential to foster trust and ensure respectful care. An HCP voiced concerns about how the lack of continuity of care could impede their efforts to build meaningful relationships with community members. They shared:

Any kind of care when it’s that broken and inconsistent with who’s providing it, you’re not building a relationship with a care provider and establishing trust and rapport and as a provider getting familiar with that patient and their story and the patient not having to retell the exact same story every time they go in and see someone new. (H1)

Another HCP shared their observations of community members who choose to seek health care outside of La Loche, noting that the community members experience a sense of mistrust toward the local health care center.

### Major theme 4: Strength-based solutions

The final theme highlighted the recommendations provided by community members and HCPs for enhancing pain management, improving pain communication, and advancing community health care services. In addition, participants suggested possible outcome indicators that could be observed to determine the level of improvement within community from future interventions. The subthemes for both community members and HCPs were (1) elements to improve pain assessment tools, (2) innovation in robotics, (3) outcome measures, (4) resources for pain management and disease prevention, and (5) recommendations to enhance care.

#### Elements to improve pain assessment tools

Participants offered suggestions on how pain assessment tools could be improved to better facilitate pain communication within the community. One participant expressed their own experiences using a pain assessment tool that was meaningful. They shared:

Well, when the doctor showed me all those, the skeleton and the pain scale with the one to nine to ten, which would be the worst and one was the least worst and stuff like that. That was good. Really simple to follow. … Like, the Dene language, our language, the majority of the language is all facial expressions. Like, one word could mean a lot with different expressions, facial expressions. (C3)

Participants also demonstrated storytelling as a way to describe their lived experiences with pain. They often recounted their pain journeys, beginning with the initial onset of pain, through to the cause of their injury and its impacts on their emotions, culture, and daily activities. By allowing adequate time and being attentive, participants shared valuable insights into their pain experiences.

Health care professionals discussed adaptations to current pain scales to better facilitate pain communication and considering the language barrier, which heavily impacts pain assessment in La Loche. They suggested utilizing more visual pain assessment tools with auditory formats of Dene descriptors and exploring ways to work around language barriers to better serve the community. One HCP said:

Yah, I think that the visual analogue scale is too abstract. … Having something that’s a little bit more concrete, and then I’m noticing some more of that storytelling and whatever descriptions of pain … maybe having something that allows the people to select something a bit more concrete, a word or face or something like that would help. Then with the language barriers, too, I don’t feel like I’m always explaining well, so having something that would help me translate certain extensive pain to other languages, to Métis to Dene, Cree, whatever people are speaking, I think that could be helpful, too. (H3)

#### Innovation in robotics

Community members suggested increasing the utilization of the remote presence robot in community. Many voiced the possible benefits of having a telehealth program within community to eliminate travel and increase availability of services in community. One participant said, “Yeah, it’s better if they start using it [the remote presence robot] on the people instead of hiding or tucked away someplace. Should bring it out and start using it on people” (C8).

Participants identified potential obstacles that could arise from using the remote presence robot, including language barriers and technological difficulties. They recommended addressing these challenges by having a nurse or translator present during consultations using the remote presence robot.

Health care providers expressed enthusiasm for the potential of utilizing the remote presence robot in the health care center, which could lead to more timely care and expanded services and reduce the need for patients to travel unnecessarily to access health care services. They shared:

…When you’re dealing with MSK issues where there’s a lot of value in in-person assessment, in-person treatment, that’s always best-case scenario. But when it’s a lengthy wait for those type of services and you can cut that wait time drastically by doing things remotely in the meantime, then that’s going to lead to better outcomes and patient satisfaction and care. (H1)

Another HCP expressed the potential benefits of limiting travel for patients seeking health care services, stating, “Like I said, it would be easier for them to come in contact with a doctor from South than travelling” (H4). Another HCP identified the potential enhancement of interdisciplinary care with use of the remote presence robot:

…You know, we do have physios in the far northwest, too, who actually have some really good backgrounds in persistent pain, … so I think that just partly through working with these populations for many years as well as different continued education pieces … linking up with other people, networking with other providers that have similar skill sets or even more advanced training in those areas certainly doesn’t hurt. (H1)

Health care providers also identified potential challenges with the use of the remote presence robot. They expressed concerns about patients showing up for their appointments over the robot, challenges associated with the use of technology, establishing a meaningful relationship over the robot, and overcoming the language barrier. They emphasized the importance of having a local staff member present at appointments alongside the remote presence robot not only to act as a translator, if necessary, but also to provide a more comfortable environment to facilitate conversations.

#### Outcome measures

Community members identified potential assessment indicators to evaluate the effectiveness of future pain management interventions within community. These included reduced wait times at the health clinic, decreased travel time for accessing health care, regular follow-ups with patients, and conducting surveys.

Health care providers recommended patient and HCP surveys to gain meaningful feedback and emphasized the importance of conducting surveys in person to offer more community engagement and relationship building.

#### Resources for pain management and disease prevention

Several community members shared that engaging in physical exercises, visiting physiotherapists, and seeing chiropractors have provided relief of their chronic MSK pain, indicating a need for accessible rehabilitation services within the community. One community member shared the importance of movement in his pain management (with the help of a translator), “He said that he basically just helps himself by doing the stretching at home and just walking around at home when he feels the pain” (C6).

Additionally, community members shared their insights on possible solutions that could help manage pain for the residents of La Loche. Among the ideas suggested were educational support groups that could improve health literacy and promote physical activity such as a walking group. Some community members proposed home visits from HCPs to aid those with pain.

Health care providers discussed current resources available in community for MSK pain management and promotion of physical activities, including physiotherapy, mental health services, addiction services, and open gym nights at the local school gym.

Health care professionals explored potential additional services that could benefit the community, including increasing the number of physiotherapy positions available, providing access to translation services, establishing support groups, offering walking programs, and implementing a telehealth program to enable access to specialists. One HCP expressed the following perspective:

I have quite a few patients that I’ve asked about walking and they’re all afraid of the dogs in town, which is a legitimate concern. I would really love to see, I don’t know what the school is doing, but a program where people could get out and do some activity within the gym. ’Cause walking is so great for you and it’s so social, it’s active, it’s good for pain management. … Just to have a safe space to do that and then it could build that social aspect as well. (H3)

#### Recommendations to enhance care

Community members provided valuable insights on how HCPs can establish trust, promote a safe environment to facilitate meaningful conversations during pain assessments, and respect traditional ways of healing. They highlighted the importance of practicing patience and actively listening to the patient. One participant shared (conversation facilitated by a translator):

He’s saying that when you go to the clinic, you sit there for more than three or four hours. He’s also saying that when a person goes to the clinic, obviously, they’re hurt. They should be examined more and be taken into real consideration instead of saying, “Oh, you’re not really hurt. You don’t look really sick. I’ll just give you Tylenol. Go home.” (C6)

Participants also expressed the significance of incorporating cultural practices into the healing process, including the use of traditional medicine. One participant shared, “Rat root, I use that” (C5). Another participant said, “Well, drinking teas and also got rubs that were made out of natural products” (C2).

Health care professionals emphasized the significance of providing culturally sensitive care, continuity of care, and respect for traditional healing practices. They recognized that trust is a crucial component of patient care. One HCP said, “I think that would increase the trust if it was a local doing that intermediate support. Yeah, I think that would be helpful. It takes a long time to build rapport in La Loche” (H2). Another HCP shared:

I guess one thing that would kind of stand out is understanding that there is lots of people that follow traditional medicine in the North and do different herbs for medicine, and not taking an overly Westernized approach in kind of disregarding those traditional practices just because they don’t have a bunch of RCT[s] [randomized controlled trials] behind them. … I think it’s important to be evidence-based practitioners while at the same time respecting the patients and their beliefs and their traditions and their cultural practices. (H1)

Additionally, HCPs shared that it would be beneficial to have a clear understanding of funding systems to help patients cover their travel costs to access health care.

## Discussion

Indigenous populations in Canada have historically been and currently are impacted by colonialism, systemic oppression, cultural and structural injustices, and interpersonal injustices, leading to health care and pain disparities. Unfortunately, chronic MSK pain disproportionately affects Indigenous Peoples in Canada. Our research collaboration with the northern Saskatchewan community of La Loche aimed to identify the community’s pain management strengths and needs through a community needs assessment. Qualitative analysis of community member and HCP interviews revealed four important themes: (1) impact of pain on daily living, (2) barriers limiting access to care and the understanding of pain between health care provider and patient, (3) systemic oppression and negative experiences with health care, and (4) strength-based solutions.

The narratives shared by community members in La Loche were echoed by HCPs, highlighting a mutual understanding of the obstacles faced by individuals in terms of pain management and health care access. Community members voiced barriers in accessing and receiving equitable care, as well as impacts of chronic MSK pain on their daily functioning. They described the systemic oppression experienced within the health care system, including being stigmatized and unfairly labeled as “drug seekers” when seeking pain management. Community members identified barriers in pain communication and expressed a sense of being unheard by HCPs. Health care providers emphasized the importance of considering worldview differences and how those may influence the perception and communication of pain, particularly when engaging with Dene/Métis community members.

Health care providers demonstrated a general awareness of the challenges faced by Dene/Métis community members in accessing health care; community members provided specific examples. The shared understanding between HCPs and community members regarding barriers to effective pain management underscored the significance of addressing these issues to enhance the quality of care provided. Despite this recognition, there appeared to be a lack of proactive measures in addressing the identified barriers.

The needs assessment revealed issues related to experiences of racism, lack of continuity of care, lack of access to services, financial barriers, inadequate pain assessments, insufficient information about care, extended wait times, suboptimal clinic models, language barriers, and travel difficulties. Access to health care is a recognized treaty right for First Nations Peoples in Canada. However, both community members and HCPs reported challenges in access to health care due to complex governmental and organizational jurisdictions. Navigating the paperwork to obtain health care and travel coverage for health care has been difficult. These findings are consistent with the outcomes of a prior research initiative led by Reichert and colleagues,^47^ focusing on the needs assessment of the communities in a First Nation Tribal Council in Saskatchewan. In that study, community members similarly conveyed instances of service delays attributed to administrative complexities and challenges related to financial coverage.^47^ Racism against Indigenous Peoples is widespread within health care systems in British Columbia and Alberta and across Canada.^19,29,47^ Historical and systemic racial biases in Canada have been entrenched within laws, policies, institutions, organizations, education, health care, the justice system, and societal values that perpetuate white privilege and create disparities for marginalized communities. La Loche community members, like many Indigenous Peoples across Canada, have described experiencing negative stereotypes such as being labeled as “drug-seeking” when accessing the health care center for pain management.^30,31^ Due to the stereotypes, Indigenous Peoples have negative experiences when accessing health care, which can lead to serious harm or even death, such as occurred with Joyce Echaquan and Brian Sinclair. Smith and colleagues^48^ noted that experiences of racism can negatively impact health through compounding negative experiences and “racial battle fatigue,” which can cause mental and physical exhaustion for individuals dealing with ongoing discrimination. Some La Loche community members have expressed hesitancy about accessing health care services at the local clinic, citing concerns about judgment and shame. This has led to individuals avoiding seeking medical help altogether or choosing to travel 6 or 7 h to urban centers for services. Consistent negative experiences and discrimination within the health care system can lead to mistrust and avoidance of accessing health services, as reported elsewhere in the literature.^19,30,31^ All of these compounding factors can influence an individual’s perspective on accessing health care and can lead to an avoidance of health care services, resulting in a higher prevalence of chronic illnesses within the community.

Community members expressed the need for comprehensive pain assessments, thorough examinations, active listening to patients’ concerns, and delivery of patient-informed care. They emphasized the importance of patience and listening to patients’ storytelling. Both community members and HCPs identified challenges with language barriers, travel barriers, long wait times, and an ineffective clinical model. They called for improvements in appointment booking, availability of translators to alleviate language barriers, and an increase in staff capacity to handle the high demand and long wait times.

There were some differences in the awareness and understanding of health care challenges between HCPs and community members in La Loche, specifically in the area of negative health care experiences. Though HCPs generally acknowledged the barriers faced by community members such as lengthy wait times and difficulties accessing non-emergent health care, they may not have fully grasped the adverse experiences that community members have encountered at the local health care center. In fact, some community members expressed preference for seeking care in urban centers and other communities. Community members reported a lack of informed decision making, confusion regarding their diagnosis and treatment plans, as well as instances of feeling unheard, experiencing shame when seeking pain management, and having their consent violated. In contrast, HCPs recognized the existence of discrimination within the health care system, but the extent of its impact on individuals was not fully understood. Overall, though there are areas of agreement between HCPs and community members in acknowledging certain challenges, it is imperative for HCPs to deepen their understanding of the negative experiences shared by community members to effectively address barriers to delivering comprehensive pain management strategies and fostering trust within the community.

This needs assessment yielded five major recommendations for improvement of chronic MSK pain management within La Loche.

### Patient-centered and community-directed care

Patient-centered and culturally responsive health care delivery in La Loche requires a tailored approach that considers the unique needs and experiences of each patient and the community. Patients should be met where they are, and health goals should be developed collaboratively with a focus on effective pain management to improve functionality in all areas of life, rather than solely on curing pain. It is crucial for HCPs to actively listen to patients, understand their experiences, and foster trust through shared decision making. A recent communities-directed needs assessment conducted in First Nations communities in Saskatchewan^47^ yielded similar findings highlighting the significance of a patient- and community-centered approach in all aspects of health care delivery within the File Hills Qu’Appelle Tribal Council communities. A community-directed approach, facilitated through the formation of a community advisory group, could enable more culturally relevant care and promote Indigenous ways of knowing. A community advisory group with local governance, Elders, and Knowledge Keepers could ensure that the community members’ voices are heard during health care center decision making.

The Royal College of Physicians and Surgeons of Canada emphasizes the importance of effective communication and patient-centered care through the CanMEDS framework, which encourages HCPs to consider not only a patient’s medical condition but also their family history, socioeconomic status, social issues, and other social determinants of health.^49^ Chronic MSK requires an interdisciplinary team with a holistic approach that considers the biopsychosocial model and recognizes that an individual’s background, socioeconomic status, and cultural upbringing can impact their experience and expression of pain.^10^ Pandey and colleagues also identified the importance of having a multimodal approach and an interdisciplinary team for chronic pain management in Saskatchewan.^50,51^ Their chronic pain clinic team consisted of pain specialists, psychiatric nurses, social workers, pharmacists, and traditional healers, which created a supportive environment and ensured patient-centered care.^50,51^ The formation of an interdisciplinary team through virtual health care could be beneficial for the community of La Loche for chronic MSK pain management.

### Clinic model and staffing requirements

Due to the remote location, HCPs often do not reside in the community, creating challenges in providing continued care. Efforts should be made to recruit and retain local HCPs committed to long-term work within La Loche. Increasing staff capacity and expanding the primary care clinic would improve access and alleviate long wait times. Current appointment booking methods at the local clinic may require review to enhance accessibility to health care. Moreover, utilization of the remote presence robot could offer enhanced specialized care for the community and the formation of an interdisciplinary team comprised of pain specialists, psychiatric nurses, social workers, physiotherapists, pharmacists, and traditional healers to address chronic MSK pain management. Pandey and colleagues have established the benefits of the multimodal form of care for residents in Saskatchewan experiencing chronic pain.^50,51^

### Practitioner education and awareness

Health care providers working in La Loche would benefit from education and experience in the community to ensure a deep understanding of community norms, the Dene and Métis languages and cultural needs, and the historical context that includes historical atrocities such as colonization, residential schools, intergenerational trauma, and the 60s scoop. It is crucial for HCPs to recognize and address racism, discrimination, and systemic oppression in the health care system. Integration of HCPs into the community can lead to a better understanding of community members’ lived experiences and foster meaningful relationships. Seeking guidance from Elders and Knowledge Keepers would help HCPs gain insights into the community and its history. These recommendations were also identified by Reichert and colleagues.^47^ It was recommended that HCPs should receive specialized training in trauma-informed care, chronic pain management strategies, and comprehensive pain assessments with proper physical examinations, which is well supported in the literature.^52^ Additionally, due to experiences with lack continuity of care, prescription review for chronic MSK pain is required to ensure that all prescriptions are reconciled. Patients using opioids should be connected to appropriate services for further assessment and management. The above recommendations align with the objectives of the Canadian Pain Task Force, which advocates for increased awareness and education on chronic pain and emphasizes the importance of specialized training for pain management.^53^

### Community education and awareness

Community members in La Loche would benefit from access to information sessions on pain management techniques, injury prevention, physical exercise, strategies for supporting those who live with chronic pain, and lifestyle modifications. Individuals should be informed of the goals of chronic MSK pain management including that it is to improve quality of life and functionality in all areas of life. El-Tallawy and colleagues^5^ found that there is a lack of health advice and education provided to patients with MSK conditions in primary care settings, with only 20% of patients receiving such support. Information sessions on racism, discrimination, and empowerment can help community members identify and address biases to help them to advocate for themselves and others. It is also important to increase awareness of local resources, including physiotherapists, mental health support, and the opportunity for care delivered via the remote presence robot to ensure that community members can access resources without travel. Additionally, community members acknowledged the possible harms with the use of opioids for chronic pain management and preferred alternative strategies for pain management such as physiotherapy and physical activities. Some community members expressed stigma surrounding the OAT program. Therefore, education and information sessions regarding the OAT program should be made available to raise awareness of its goals and create a safe environment for program participants. This may help to eliminate negative stereotypes that may stem from misinformation about the program. Presently, University of Saskatchewan researchers are collaborating with La Loche community members and Dene/Métis language experts to create pain materials in Dene and Michif.

### Community resources

Creating a safe and accessible space for physical activities, such as a walking facility, would promote physical wellness and allow community members to engage in activities without concerns for safety. The formation of support groups for those living with chronic pain would offer social support and promote a sense of community. It could also provide a safe and inclusive environment for individuals to share their experiences and connect with others. A peer support group was also identified by Pandey and colleagues as a recommendation suggested by participants who were part of a chronic pain clinic in Saskatchewan.^51^

The recommendations made align with the goals of the Canadian Pain Task Force^53^ in the areas of “increase awareness, education, and specialized training in pain,” “improve access to timely, equitable, and patient-centered pain care,” and “ensure equitable approaches for populations disproportionately impacted by pain.” Furthermore, the recommendations also align with the Truth and Reconciliation Commission of Canada’s Calls to Action, specifically numbers 22 and 23.^54^ These calls emphasize the importance of working in collaboration with Elders and Knowledge Keepers within the health care setting, as well as providing education to HCPs on cultural sensitivity and competency.^54^ Through the implementation of these recommendations, an environment that is inclusive and culturally responsive could be developed, encouraging community members to become advocates for their health and providing them with the necessary tools to self-manage their pain more effectively.

## Limitations

This needs assessment was conducted in close partnership with the community of La Loche, and the resulting recommendations are specific to the Dene/Métis members of this unique community. As such, the findings of this project may have limited generalizability to other Indigenous communities. However, the collaborative approach and outcomes of this project may offer valuable insights for future research in similar contexts. Additionally, participants mentioned challenges with the OAT program in the data; however, the objective of the study was not to explore the impact of opioid-related harms on community members.

## Conclusion

The needs assessment conducted in collaboration with the community of La Loche, a Dene/Métis community in northern Saskatchewan, provided valuable insights into chronic MSK pain management. Through conversations with community members and HCPs, recommendations were made to improve communication and support for community members living with chronic MSK pain. The findings emphasized the importance of community engagement, cultural responsiveness, and anti-racism/anti-oppression practices to enhance chronic pain management in La Loche. The implementation of these recommendations would support an inclusive and culturally responsive environment, empowering community members to become advocates for their health and providing them with the necessary tools and support to effectively manage their pain. The successful implementation of these recommendations could also enhance the quality of health care services provided in La Loche.

## Supplementary Material

Supplemental Material
