# Supplementary material for: Understanding Needs for Chronic Musculoskeletal Pain Management in a Northern Dene and Métis Community: A Community Based Needs Assessment
Source: Can J Pain. 2024 Dec 6;8(2):2412560. doi: 10.1080/24740527.2024.2412560 (PMC11633199; doi:10.1080/24740527.2024.2412560)
Supplement: Supplemental Material [file UCJP_A_2412560_SM1030.docx]

# **Appendix A- La Loche Community Participant Focus Group and Individual Interview Guide**

***Please note that these questions will be reviewed and further informed by our community partners… as they may have other suggested questions/modifications to questions***

**Preamble:**

Hello, my/our name(s) is/ are X and my pronouns are (she/he/they), and we are part of the research team looking at your community’s needs for chronic pain related to bones, joints and muscles. Chronic MSK pain includes pain from muscles, bones and joints that has been present for at least 3 months or longer. We are also interested in how the robot at the community health facility can be used to join chronic MSK experts from other locations with your community members to help in their chronic MSK care (virtual care).

Thank you for agreeing to talk to me/us about your experiences with having chronic MSK problems.

As you know from the consent form we just reviewed together, the information you provide will help us to better understand: 1) your experiences of living with chronic MSK pain; 2) what your unique preferences might be regarding the use of technology, specifically a remote robot technology, to support chronic MSK pain care; 3) what type of information would help to understand if using robot technology is helpful in the treatment of people with pain in your community.

This information you provide will help us to better understand how the health system can work better for you, for treating and living with bone, muscles or joint pain, which may also be relevant to other members in your community. What you share with us will help design and evaluate a process for using remote robot technology as well as in-person care to treat pain in your community, with the hope of improving access to physical therapy and pharmacy services.

We hope this information will be used to develop further health services and supports to benefit the community.

This interview/ discussion should take approximately 30 minutes for one-on-one interviews, and up to 2 hours for group discussions.

I/ We will be recording our conversation today and the recording will then be typed up into a written script for analysis after the research project is completed. Are you ok with proceeding with the interview at this time?

Do you have any questions before we get started?

If no…

Let's get started…I/we am/ are turning on the recorder now.

1. **Tell me/ us a bit about your chronic MSK pain.** (*pause after this statement and wait for a response; and if needed, use the following bulleted list as probes to draw out information from the participant)*
   1. How long have you experienced this problem or problems?
   2. How has having pain affected your life?
      1. Physical functioning (intense activities, moderate activities, dressing and bathing daily activities)?
      2. Social participation? i.e. activities with family, friends, community, work
      3. Emotional consequences?(feeling tense, depressed, having enough energy)
      4. Your ability to practice your culture and/or spiritual activities?
      5. Other? (e.g. sleep)
2. **Can you describe to me your behaviour when you are in a lot of pain*?*** *(if needed, use following as probes)*
   1. How do you communicate to others?
   2. How do you handle daily tasks when you are in a lot of pain?

*(Examples to describe if needed)*

- 1. Get frustrated more easily, get quieter, cry, look for distractions, groan, go lay down, etc.

1. **Tell us what it is usually like to communicate with health providers about your pain.**
   1. Do you find it difficult to talk about your pain or explain your pain?
2. **Is there anything that would make you more comfortable to talk about your pain?**
   1. How does the environment affect how you feel?
   2. Is there anything the health provider can do to make you feel more comfortable when talking about your pain?
3. **How have you described pain to health providers in the past?**
   1. Verbally?
   2. Demonstrated the location?
   3. Used certain pain scales that required you to compare your pain to faces, colors or numbers?
4. **If you have used pain scales to talk about your pain to your health providers, how well do you feel they allowed you to communicate about your pain?** *(If needed, use probes*)
   1. Challenges understanding language?
   2. Challenges understanding of the meaning of the pain scales?
   3. Cultural limitations?
5. **If you have used pain scales, what did you think about them?**
6. **What would make pain scales better or easier for you to use to explain your pain?**
7. **What types of supports/ services have you found helpful for management of your pain?**
   1. Health care services? (including physical therapy services…? Medications?)
   2. Community supports?
   3. Local/traditional cultural practices?
   4. Others? (e.g. companion animals, other self-management strategies?)
8. **Can you tell us about any challenges you have had in trying to access health care for your pain issues?**
   1. Travel?
   2. Wait times?
   3. Financial/ costs?
   4. Cultural?
   5. Comfort interacting with health care professionals?
   6. Language or interpretation? (cultural languages, use of pronouns or reference to incorrect gender, etc).
   7. Mobility accessibility?
   8. Experiences with racism?
   9. Other?
9. **What types of services and/ or supports do you think would help to support you in better treating and managing your pain and overall abilities to do the things you want to do?**
   1. What considerations for new services and providers should be taken into account specifically regarding your language and culture, the culture and protocols in your community, that new providers should be mindful and aware of to ensure care provided is culturally appropriate and responsive to your needs and preferences?
10. **Do you think that using technology like the (remote presence) robot might help people in the community access better care related to their pain?**
    1. Why or why not?
    2. What might be some important ways to ensure that your culture and perspectives are respected and honored while using robot technology?
    3. What do you see as potential challenges to using robot technology to treat pain problems in your community? (these could be anything including language or other cultural practices… as examples)
11. **Do you think that linking community members, such as yourself, to physical therapists and pharmacists using remote technology and with the support of local health care providers would be helpful for people in the community with chronic pain problems?**
    1. Why or why not?
    2. If technology could be used to increase access to care (such as physical therapy and pharmacy care) to treat low back pain in communities such as yours, what do you think is needed to ensure that the process and interactions with health care providers are meaningful to you?
12. **How would we know if this type of service were relevant and useful for people living with pain in your community?**
    1. Do you have some ideas on what types of measures would help capture the usefulness of using robot technology to increase access to physical therapy and pharmacy care?
       1. Participant experiences/ stories?
       2. Less pain? (Measuring pain levels?)
       3. Better quality of life? (Clarifying what types of activities you are now able to participate in, that you couldn’t do before receiving physical therapy care via robot technology…?)
       4. Better movement/ mobility?
       5. More able to participate in social/ community activities?
       6. Less use of prescription medicines?
       7. Less travel from the community?
       8. Others?
13. **Is there anything else you would like to share with us about either your experiences with pain problems, health care access/ use or what type of new service using the robot or other supports in your community might be helpful?**

Thanks so much for your time and your thoughts. Before we finish today, I would like to go back to the consent form briefly. Now that we have been through the interview and you know what you have shared with me, I just want to go back though the sections we checked off and see if you still consent in the same way as before we started. It’s perfectly OK to change your mind on any of this. [At this point confirm all the check box decisions with the participant]

# **Appendix B- La Loche Health Provider/ Manager Interview Guide**

***Please note that these questions will be reviewed and further informed by our community partners… as they may have other suggested questions/modifications to questions.***

**Preamble:**

Hello, my/our name(s) is/ are X, and my pronouns are (she/he/they) and we are part of the research team looking at community needs for chronic musculoskeletal (chronic MSK) pain management using the remote presence robot and health care teams.

Thank you for agreeing to talk to me/us about and share your guidance and experiences.

The information you provide will help us to better understand: 1) your observations of experiences of community members with chronic MSK pain; 2) what your preferences might be regarding the use of remote presence robot technology to support care; 3) what type of measures would be relevant to you and/ or the community to help understand if a new health service using remote presence robots and health care providers teams is useful.

This information you provide will help us to better understand what the community needs for chronic pain are and help design and evaluate a pilot intervention for people in the community with chronic MSK pain.

Our hope is that this information will be used to develop further health services and supports to benefit the community.

This interview/ discussion should take approximately 30 min - 1 hour.

I/ We will be recording our conversation today and the recording will then be typed up into a written script for analysis after the research project is completed. Are you ok with proceeding with the interview at this time?

Do you have any questions before we get started?

If no…

Let's get started…I/we am/ are turning on the recorder now.

1. **Tell me/ us a bit about what you have observed regarding people who experience persistent chronic MSK pain?** (*pause after this statement and wait for a response; and if needed, use the following bulleted list as probes to draw out information from the participant)*
   1. How has having chronic MSK pain affected the lives of people in your community?
      1. Physical abilities?
      2. Social participation? i.e. activities with family, friends, community, work
      3. Emotional consequences?
      4. The ability to practice culture and/or spiritual activities?
      5. Other?
2. **What Western pain scales have you traditionally used to assess Indigenous patients’ pain? In your opinion, how effective has that process been in the past?**
3. **From your experiences, which types of pain scales were the most preferred by Indigenous patients when asked to share their pain experience?**
4. **Describe your experience communicating about pain with patients when using the Western pain scales.**
5. **In your opinion, is there anything we could do to improve pain scales?** *(Prompts if need)*
   1. What did you like or didn’t like about the scale?
   2. What things do you think would make pain scales better for your patients?
6. **Do you have any additional concerns about your ability to understand pain from an Indigenous patients’ perspective?**
7. **What types of supports/ services do you think would be helpful for management of chronic MSK pain?**
   1. Health care services (including physical therapy services…? Pharmacy services? Medications?)
   2. Community supports?
   3. Local/traditional cultural practices?
   4. Others? (e.g. companion animals)
8. **Can you tell us about any challenges people in the community have reported or experienced in trying to access health care for chronic MSK problems?**
   1. Travel?
   2. Wait times?
   3. Financial/ costs?
   4. Cultural?
   5. Comfort interacting with health care professionals?
   6. Language or interpretation? (cultural languages, use of pronouns or reference to incorrect gender, etc).
   7. Mobility accessibility?
   8. Experiences with racism?
   9. Other?
9. **What types of services and/ or supports do you think would help to overcome these challenges?**
   1. What are community supports and strengths in the community that would help to support people in the community with chronic MSK pain?
   2. What types of information, cultural understanding and communication is important for new providers to have and demonstrate, to ensure care is culturally responsive, appropriate and patient centered?
10. **Do you think that using technology like the remote presence robot might help people in the community access better care for their pain problems?**
    1. Why or why not?
    2. What do you see as potential challenges to using robot technology to treat musculoskeletal problems in this community? (these could be anything including language or other cultural practices… as examples)
11. **Do you think that a new service linking local health care providers with remote health care providers like physical therapists and pharmacists would be helpful for people in the community with chronic pain problems?**
    1. Why or why not?
12. **How would we know if a new type of service were relevant and useful for the community? What types of measurements would help capture this?**
    1. Participant experiences/ stories?
    2. Less pain?
    3. Better quality of life?
    4. Better movement/ mobility?
    5. More able to participate in social/ community activities?
    6. Less use of prescription medicines?
    7. Less travel from the community?
    8. Others?
13. **Is there anything else you would like to share with us about either your observations of people with back problems, health care access/ use or what type of new service using the robot might be helpful?**

Thanks so much for your time and your thoughts. Before we finish today, I would like to go back to the consent form briefly. Now that we have been through the interview and you know what you have shared with me, I just want to go back though the sections we checked off and see if you still consent in the same way as before we started. It’s perfectly OK to change your mind on any of this. [At this point confirm all the check box decisions with the participant]

# **Appendix C- Additional Quotations from Semi-structured Interviews with Community Members**

**Table 2: La Loche Community Members Table of Quotes**

**1. Major Theme 1: Impact of Pain on Daily Living**

In this table community member participants are represented as “C” and the number refers to the specific community member interview from which the quotes are taken.

| **Subthemes** | **Sub-subtheme** | **Quotes** |
| --- | --- | --- |
| **Pain Affecting Culture** |  | Like I said, we use wood stove. We- I can't help him get wood, cut wood because I'm in the same pain as he is. [C4] |
|  |  | For sure. I can't dance or anything. I would dance…[C1] |
| **Pain Affecting Emotions** |  | Well, it makes you kind of depressed cause you're always stuck at home, or you can't enjoy the activities you use to do, even walking, that simple. [C2] |
| **Pain Affecting Physical Activities and Daily Living** |  | It does. It affects my breathing like the pain I have in my back causes my- it's so much pain that it gets hard to breathe when I sleep and when I wake up. [C5] |
|  |  | I used to get up at six, seven o clock in the morning and because the pain comes around in the morning when I’m sleeping, I have to get up. that’s when the pain goes away. [C8] |
|  |  | Some days, it's hard for me to get into a shower. It's hard to do my house chores or anything. It varies. I can't walk on it. I have a hard time walking and I have to wear a tensor bandage. [C2] |
|  |  | We have had to hire people a few times- we both had to hire people to do stuff for us around the house. He has to hire some guys to cut wood for us, and me because I cannot clean up or whatever, I have to hire a girl, some young girl to do my housework for me. That wasn't us, we were perfectly good people where we could just get up, 7 o clock in the morning to go to work..  I used to get up at five o clock in the morning cause I had to go to work at seven until seven in the evening, a 12 hour shift. I did that no problem, I can't do that now. There's no way I'm gonna get up at 5 o clock in the morning, jump in the shower, get on the bus at 7 to get to the office to go to work and sit there all day till seven in the evening. [C4] |
| **Pain Affecting Family and Social Participation** |  | I don’t do a lot of stuff that, how people do, like bending over, sitting with kids. I can’t do that eh. I can’t sit long. [C7] |
|  |  | I don't know how to — or I can't lift anything too heavy anymore. Bending, I notice too, just carrying my nieces on my back or something like that, whatever, doing tasks like that, will start affecting my back. [C1] |

**2. Major Theme 2: Barriers Limiting Access to Care and the Understanding of Pain between Healthcare Provider and Patient**

| **Subthemes** | **Sub-subtheme** | **Quotes** |
| --- | --- | --- |
| **Communication about Pain** |  | Yeah, I usually point to where it hurts. [C5] |
| **Language Barriers** |  | Like a language barrier for sure in this community because a lot of Elders are Dene speakers so that might be a challenge for them to get their point across. [C2] |
| **Limited Health Resources and Long Wait Times** |  | We don't have a lot of doctors in our community. We only have like two or one. We only have a limited walk-in clinics so it's harder for them to see the doctor and get the help that they need. Let's say they're in pain, they'll be lucky to get in because there's only limited doctors and stuff like that. [C6] |
|  |  | If you went there to see a doctor or nurse, like what he said, unless there's more than six people or whatever, the doctor won't come in. They'll tell you "unless we have this many people we are not gonna call the doctor." I said "hello, what do you mean? I need the doctor to see this, and then they keep insisting that they're not gonna call the doctor unless there's six or more people.” [C4]  Sometimes it gets to the point where we have to call relatives or friends to come into the clinic just to register their names so that the doctor can come. Now is that right? [C3] |
|  |  | These doctors in town here, they're not considered full time, they're considered locums, we can’t sign the contracts, that's one of the main reasons what we can't get help from the doctors. Because they're not considered permanent, they’re considered locums…[C3]  They're just transient doctors. [C4]  Totally, yeah. And you go see a different doctor and they say ‘wow, you've seen a different doctor, I can’t help you. [C3] |
| **Traveling for Care** |  | I usually go to a walk-in health clinic in P.A. or in Saskatoon. [C2] |
| **The Need for Enhanced Education** |  | At first, cause I was a cancer patient, I was put on some pump medication when I was in the hospital, I didn’t know what it was, morphine? The doctor says, ‘if you have pain, just press it.’ So when I had pain I did that. I've never experimented with drugs in my life, ever. So when that thing got me smiling, happy and everything, I kinda thought ‘maybe that's something I shouldn't- whatever.’ Anyways, once I was discharged from the hospital I was given some pain meds and I was on pain meds for a while, but then the doctor just doesn’t give it to you anymore because of some people, you know? It makes my life hard cause my pain is really bad and I don't have nothing to manage it with, so I just use Advil, Aleve, whatever works for the pain. So that's how my pain really affected me, it still does. [C4]  If I was on my feet for two days, I gotta stay with my feet elevated for three days because my feet get so fat, my legs. I go to the doctor, they say ‘your body's retaining water.’ Well is there a treatment for it? Is there something wrong, why is it doing that? Nothing, they just tell you ‘your body's retaining water. [C3] |
| **Financial Barriers** |  | Financial resources as in money, vehicle transportation, no support. I'm currently learning how to drive in the city. I'm treaty, so I think that would cover some medical things. Other than that, I get no support. I go to school and get the minimum wage of seven and that goes straight to my vehicle which needs parts and [it goes to rent]. So, there's no money left. [C5] |

**3. Major Theme 3: Systemic Oppression and Negative Experiences with Healthcare**

| **Subthemes** | **Sub-subtheme** | **Quotes** |
| --- | --- | --- |
| **Assumption that Pain Means Seeking Drugs** |  | First time I start seeing the doctor about my shoulder, I was asked if I could get, like ‘What kind of medication do you want?’ You want the Tylenol 3? That’s what they asked me. The doctor should describe what pills I should get not what I want. I was asked one time. I didn’t like that. I wasn’t there for pills. [C8] |
|  |  | I don't bother going to the hospital here because my other doctor was in Saskatoon. I've been living here now for six years but I don't even go to the hospital….They kinda look down on you and treat you different and they say right out, ‘You're just here for drugs,’ so I don't even bother going there. [C2] |
|  |  | It's not just about getting people to talk while they're here, it's not just about getting high, we're trying to control our pain so we can sustain our livelihood, practice our culture. You can't do that when you're in pain, you can't even think about anything when you're in pain. If you go in and say you're in pain, you say you're looking for pain medication,- [C3]  They say that to you. ‘You're just looking for pain meds.’ And that's not right, that's very discriminative. [C4] |
| **Patients Feel Unheard** |  | Yes, health providers are not listening to [him], not to just him, he also say that to the people. Most people, when you go into the hospital, you just get sent home with Tylenol. It's not fair. …They will still not take him seriously. He also just said that they won't do anything anyways even though we tell them where it hurts and all that…They just don't give out any other medications when you ask for help or they don't offer any other service besides getting seen, not getting fully examined, and offering Tylenol. That is it. [C6] |
|  |  | The doctors, they just tell me that I'm fine. Yeah. They think like, just because I look healthy, that I'm okay. But I'm not. I usually go there and they tell me I'm healthy; they don't really look at me; they don’t do anything. [C1] |

**4. Major Theme 4: Strength-Based Solutions**

| **Subthemes** | **Sub-subtheme** | **Quotes** |
| --- | --- | --- |
| **Elements to Improve Pain Assessment Tools** |  | Okay yeah it seems lockup. All my shoulders, my elbows, everything. Like right from the knees. So, what I used to do, back in the day in the sports, I hit the boards in the back first, so there used to be a Saint-Paul Hospital in La Loche. It used to be on Saint-Martin or something—forgot the name—but, I hit the boards there. I laid in bed for 12 days without moving. It was bruised up or something. They could do nothing. So, I got up that problem from hockey, and then my collarbone was broken. And then I used to see a chiropractor every week. there used to be a chiropractor in La Loche, and then he left. And we had nothing. I stopped going there and I started doing what he showed me to do just by myself, tried to get the pain away and all that. And then I got hit by a vehicle. My forearm broke, just one bone. Everything locks up all over. But with operating on that doesn't help, everything my hands, my knees, my toes. It's always stiff. I do a little bit of stretching, a lot of walking. When I'm home a lot of physical work, I’m a mechanic and carpenter and all that. To ease the pain. Cause I'm always moving. Other than that, I just rub myself wherever it hurts to get the pain away. [C7] |
|  |  | Like my shoulder pain it’s really painful, it comes around just for few seconds that’s it. It comes around and it goes, big gaps in between hey? That’s how it is for my shoulder, because I damaged my shoulder, when I was mining back in early 1990s. And it bothers me now. I've been seeing a doctor about it a few times and now for the last couple of months, I've been waiting to see a bone specialist, I think. That’s what I'm waiting for right now. [C8] |
|  |  | I don't know, my legs give out or a nerve — there's like nerve damage or something. My back hurts a lot. When I try and stand for long periods of time or I'm sitting a certain way — my hands, I can't do much with them either because they have arthritis; like really bad joint pain. Sometimes my body just feels inflamed... and it's sore to do daily things... My knee, I had fell one time on a cement pad and it shattered my knee cap. I went to try and get something done and stuff but had a harder in the North. My lower back, I had gotten into a car accident a while back and became unconscious, so, I didn't go in for a check up. But my quad accident shortly after that affected my back 'cause I about flew right off the quad. And my hands, I have — they're just dissociated or something on my fingers. It's hard to do certain tasks I’ve done every day. [C1] |
| **Innovation in Robotics** |  | There would have to have somebody here that has patience to explain to us, this is how its gonna work. Stuff like that. I just can’t be, for me I can’t understand the robot. I can’t even type on computer…[C7] |
|  |  | You know, people talk about it and I've seen it standing in the corner but I've never actually seen it work. I heard about one scenario where the doctors themselves were on the robot, they did a really good diagnostic on the person and the person survived….the person pulled through with help from the robot, the doctors down south. That would even be another option of seeing a pain specialist or whatever, to go to the doctor or sit in the office and look at the robot doctor…Well give it to a doctor down south who’s willing to deal with pain management that are part of a survey like this. It'll save you the time and the finances of going south, you can do it right here at the clinic, if the doctor or the clinic took part and says ‘okay Friday, we're gonna designate all the pain management, all the pain specialists to be on the robot to talk to the patients.’..Instead of, you're in pain, you're sitting in a medical taxi with 10 other people bouncing around, that's still an uncomfortable feeling. Whereas of you can talk to the doctor in the robot, go home and relax on the couch. And then the robot, you can get the pharmacist on board and talk with the doctor, you know? [C3] |
| **Outcome Measures** |  | Maybe taking a survey with the members, ask them if it's helpful. [C1] |
|  |  | They would have to do a review. [C2] |
|  |  | The person receiving the care would also have to update their status if it's pain management or if it's working or not, right? [C2] |
|  |  | It’s better if they come back and check up on the patients to see how their experience was with the robot machine. Instead of just thinking that "Oh they use the robot that’s good" and then let’s just move on to another one. Instead check up on the patient again, to see where they’re at. [C8] |
| **Resources for Pain Management and Disease Prevention** |  | Maybe a walking facility like, just a little walk course or something. Ten minutes. People don’t know how to work with time I guess I don’t know. Some people like they’ve gotta work from nine to five. Ten minutes at a time [of walking], it helps…But they don’t even like, they got nothing here, not even for a walk around the building or anywhere, right. [C7] |
|  |  | When I'm home [I do] a lot of physical work…To ease the pain…cause I'm always moving. Other than that, I just rub myself wherever it hurts to get the pain away. When I was in McMurray, Alberta, working, I used to go swimming there to keep the muscle moving. And all my muscles are moving. I swam for like an hour, every time I work up there it’s two weeks, One week out. So, 13 days out of 14 days, I used to swim every day for an hour. That helped. [C7] |
|  |  | Maybe where someone would come into the community and a group of people would participate in certain activities to help manage different sorts of problems. [C5] |
| **Recommendations to Enhance Care** |  | A doctor that understands the patient and take the time to listen to their patient instead of deciding on their own. ‘No this is wrong with you, that's wrong with you.’ A person knows their own body. [C4]  …you can talk to somebody that's knowledgeable and maybe somebody that's not afraid to try and speak the Dene language or whatever so you could feel comfortable talking to them. [C3] |
|  |  | Yeah. I like my doctor in P.A. and she's a young woman and she's really smart and she does really try to get to the bottom of everything; she'll check you for everything… She's more open with ex drug users. I have a lot of nerve damage probably from drug using. At least since I've been clean, I've been like noticing that I have nerve damage and stuff and she was really educated. She would educate you on it. [C1] |
|  |  | Unless somebody from the Saskatchewan Health Authority comes down here and disguises himself and sits there with a couple, with three, four Indians and sits there in a waiting room and then goes and sees what actually happens, is being said to the patient, like "you shut up and sit there. The doctor will come but if not, just sit there." I thought I talked to you, and if you say something, they'll call the cops on you. The cops will escort you out. [C3]  Yeah, it'll be different. The discrimination is too strong in that place. [C4] |

# **Appendix D- Additional Quotations from Semi-structured Interviews with Healthcare Providers**

**Table 3: La Loche Healthcare Providers Table of Quotes**

**1. Major Theme 1: The Impact of Pain on Daily Living**

In this table healthcare provider participants are represented as “H” and the number refers to the specific healthcare provider interview from which the quotes are taken.

| **Subthemes** | **Sub-subtheme** | **Quotes** |
| --- | --- | --- |
| **Pain Affecting Culture** |  | The things that would prevent people from doing would be most common recreational pursuits might be just like going for walks, berry picking…[H1] |
|  |  | A lot of it seems to be quite social as well like, ‘I wish I could go quadding with my friends, but I can't. Because my chronic low back pain just can't handle that.’ Or canoeing like, ‘I can't sit in the canoe for that long.’ I haven't heard as much on specific cultural things that people can't do, but those waters get muddied a little bit where I don't always know what's cultural and what's just an activity. Sometimes people will be like, ‘I can't go hunting anymore,’ and I don't know if that's a cultural tie to hunting that they're missing or whether they just liked that activity as a social outlet and it's not so much related to cultural practices. [H3] |
| **Pain Affecting Family and Social Participation** |  | Going for walks and visiting people, limited in those kinds of regards. Absolutely. From my experience it's been more about missing out on time with friends and family, not having the energy for that, being able to go to those activities. [H3] |

**2. Major Theme 2: Barriers Limiting Access to Care and the Understanding of Pain between Healthcare Provider and Patient**

| **Subthemes** | **Sub-subtheme** | **Quotes** |
| --- | --- | --- |
| **Communication about Pain** |  | The storytelling component is pretty big. I find that people’s score really high in situations when I wouldn’t necessarily consider, with how they’re presenting, to match with that high of a scale. Sometimes it gets a little bit more accurate, or what I feel is accurate, what I feel matches up with their physical appearance and presentation in that regard. It’s just really different, it’s been taught to me differently then somebody who’s First Nation living in a northern community. I feel that disconnect sometimes. Looking at somebody and they’re saying they’re a seven out of ten pain, and you look like a three to me, like there’s clearly something different in how we’re processing pain and understanding where that scale is, how to express it. I don’t think I always do a great job of figuring out what the differences are. I’m trying. One of the things that I noticed is I’ll ask for where somebody’s pain is and I’m expecting a word or an indication of a more specific spot, but I’m getting quite general answers. Then when we dig into it deeper it turns out the pain is fairly specific. I think that’s a difference in how I as a white person and potentially they as First Nations people conceptualize pain as well. I’m still trying to figure out what I think about that, what it means. [H3] |
| **Language Barriers** |  | Usually just [use] a verbal analogue scale, 0-10. [It’s] medium effective. I think where it is ineffective is when you’re dealing with Dene first language patients, and they don’t really entirely comprehend the scale. Language would be the only thing that stands out to me as a bit of a barrier when understanding pain specific to the dene community. [H1] |
|  |  | There are many people in La Loche who do not speak English or English is their second language. I actually didn’t think that was a barrier, but it can make it very difficult especially our Elder population, a lot of them don’t speak English. We do utilize our community outreach education worker to help translate as much as possible, but I would definitely say out of all the communities within our northwest region that where we’re going to see most language barriers for sure is in La Loche. It’s interesting because when we have resources that are in the Dene language most individuals don’t know how to read the Dene language, they just speak it. So, any resource really needs to be quite visual with pictures or audible we did get – I’ve seen resources in my previous position that were made in the Dene language, and no one could read them. [H2] |
| **Limited Health Resources and Long Wait Times** |  | And the last thing I was going to say is it's again a capacity issue but like if our physiotherapist could be out in community more you know they're living in [extracted for privacy] so they're often involved in activities within the community promoting physical activity and what not in those communities but we don't have that in La Loche we just don't have that capacity. [H2] |
|  |  | It's this un-serviced, it would be nice if we could have more physios up there….[H3] |
|  |  | Patients who are in chronic waitlists who have had back pain for example, 5 years, they're often not seen very quickly when we're not fully staffed such as right now and those people tend to wait sometimes very long periods before being seen. Someone who just had a knee replacement or stroke, they're going to be seen within a week or 2 whereas someone on that chronic waitlist depending on staffing levels, they could be over a year. I think on the mental health side there’s always improvements that can be made there for regular mental health patients but also patients with persistent pain on the mental health side whether that’s from a CBT [cognitive behavioural therapy] approach to managing pain which I think would be really helpful which isn't really available on offered in La Loche or very much of the northwest. On the behavioural therapy, I think that could be very useful for some patients but there’s just little to none of it offered by mental health in the northwest to just managing various psychosocial issues in patients' lives which can predispose people to developing chronic and persistent pain. [H1] |
| **Traveling for Care** |  | People that are on social assistance, they're able to get on taxis. Those that aren't on assistance or aren't Treaty, their trips aren't covered. People have more difficult time to get South if they're unemployed or employed but making, say minimum wage…As well as our seniors. We're talking about chronic pain, there's a lot of seniors with different illnesses or issues. They're not covered to go on a medical taxi so family, or they have to find a way down for their appointments for themselves. [H4] |
|  |  | Yah, travel is a hot mess. Medical taxi system right now is especially just not doing a good job. I'll book patients in and the taxi drivers will just decide not to work that day, and then my patients can't come whether they want to or not. That's tricky. Then coordinating that travel arrangement sometimes can be pretty frustrating or difficult. …Sometimes the weather inhibits people from coming whether they have a ride or not. [H3] |
|  |  | There are also a few neighboring communities outside of La Loche they're smaller but there are individuals that live there and that would be a financial barrier because that’s a lot of gas to get to town but that would be the nearest place to receive that service. The physiotherapy services offered outside of La Loche are free to the public, but transportation can cost a lot for people. [H2] |
| **Present Environment Surrounding Pain Care** |  | For pain management people will come to the Health Center clinic or the emergency room…Booking an appointment at the clinic is very difficult. You have to show up in-person or call in at 9:00am to see a physician. Spots are full by 9:10am. [H5] |
|  |  | I would say that it seems like there’s quite a few people that seem to have these ongoing pain issues... 20 years’ worth of pain...it’s been a really long time. So far in my experience it’s always been a really traumatic event that kicks off the whole cycle. It does sound like most people received some kind of medical intervention early on. Whether the revolving door practitioners that visit La Loche just breaks down that continuity of care. The people just see these one-off people coming and assume that they’re drug seeking for whatever reason. They might assume that. When you’re trying to get follow ups on things and it’s a different person every time and they don’t know what the last guy did. Sometimes referrals for imaging will get mixed up and somebody won’t know that the next guy supposed to follow through on that. [H3] |
|  |  | So, let’s say they weren’t trying to access physio directly, they were trying to access maybe their primary care provider and then moved to getting a referral for physio. So, if they were going that route, there is definitely a barrier to accessing non-emergent healthcare in La Loche so there’s an unfortunate model at the clinic…now I believe they have it where everybody needs to call each morning, they do not book ahead, because there were problems with no shows…we have trouble booking morning appointments in La Loche, it’s almost like it’s the circadian rhythm in that community starts later in the day and so afternoon they’re usually heavily booked. Whereas mornings are a lot of no shows there are a lot of people that aren’t able to make it. [H2] |

**3. Major Theme 3: Systemic Oppression and Negative Experiences with Healthcare**

| **Subthemes** | **Sub-subtheme** | **Quotes** |
| --- | --- | --- |
| **Discrimination** |  | And I also think about I don't know this for sure, but you know what about the individuals who are in pain and perhaps do need medication for that pain but are really discriminated against because of their identity? [H2] |
| **Indigenous History and Intergenerational Trauma** |  | …you have a lot of health care professionals coming to the north and really unfairly treating the community. [H2] |
| **Mistrust between Community Members and Healthcare Providers, Healthcare System** |  | I feel like the few people that I've dealt with that have had these long-standing chronic pain issues, feel really disregarded by the health care system. I think there still is quite a bit of mistrust between the First Nations and health care relationship. I had a couple interactions with people where they were just like, ‘I don't go to the doctors anymore…’ Especially people with   chronic pain. It's just so exhausting on its own, never mind to have to defend yourself and explain yourself over and over again. [H3] |
|  |  | ..there is a huge mistrust between the community and the healthcare system... So, a lot of individuals who do have access or are a little bit more privileged will choose to access services such as physiotherapy in other urban centers and are willing to drive to receive those services. [H2] |

**4. Major Theme 4: Strength-Based Solutions**

| **Subthemes** | **Sub-subtheme** | **Quotes** |
| --- | --- | --- |
| **Innovation in Robotics** |  | Yeah, I think one thing is not a fear of technology, but it can be difficult to develop rapport even in-person but sometimes if you add technology in there like a robot or a screen and now it can be even more difficult to develop that rapport. I think it would be helpful if there was a local person there that they trusted as well. I'm just a little concerned about the rapport building piece. [H2] |
|  |  | Getting patients for in-person appointments is difficult, getting them in for telehealth could potentially be even more challenging so it would be good to have a good system of reminder phone calls setup, making sure it’s extremely clear where they need to go; when they need to go, what they need to bring to that telehealth appointment. I mean the technology component itself I’m not too sure how complex that is but making sure that there’s simple instructions, maybe a staff on hand to help with setting it up, making sure that the people who aren’t as tech savvy you know Elders or non-Elders don’t have much exposure to telehealth or technology they don’t have any issues going in the room and being confused and not having things set up ahead of time. [H1] |
| **Outcome Measures** |  | Getting feedback from the people that are participating in those services would be the best place to start. But you would have to do it in-person, any type of assessment measure that's involved with a community member has to be done with them face-to-face while they're already in your center; relying on email communications or mailouts, or even phone calls. I don't know that you would get the response volume or whatever that you would need. It would be much better to do it all in face-to-face interactions. [H3] |
| **Resources for Pain Management and Disease Prevention** |  | I don't actually know. I know that I could do a better job of knowing some of those services. I know there's mental health and addiction services that people with chronic pain can certainly access. Then there's our physio services. There's the clinic in town and obviously a more emergency stuff, but beyond that I don't think I know about much about what kind of community programs are offered up there. [H3] |
|  |  | We do have a physiotherapist that come in three times a week…Plus, we have…an occupational health therapist. So, we have those. Besides different programs- I know at the school, they have open gym nights or the weight room where people can actually go just to get exercise in. [H4] |
|  |  | Individuals come in to see physicians seeking medications for pain management, while other Individuals come in to see physiotherapists for pain management where they are taught to do muscle exercises. There's nothing other than the two I mentioned. There are no facilities or a place for exercise equipment or people to go to. [H5] |
|  |  | We require a lot of Services. I believe people can benefit from having staff that can do translations, have more telehealth sessions or utilize the Robot for specialist examinations. [H5] |
| **Recommendations to Enhance Care** |  | People are okay about bringing themselves to appointments though, and there's more funding out there then what I'm aware of. I think that could be something that, help me out anyhow, is understanding better the funding systems that would help people get travel. I know to contact specific travel coordinators but sometimes my patients will show up with forms to fill out and I have no idea what organization they're going to or what kind of reimbursement they're getting. If I had a better concept of that I could potentially be advocating for other arrangements for travel other than just this one medical taxi system that I know about. One thing that I didn't mention at all is that a lot of my chronic pain people have come through SGI as well, through that system. Navigating that has also been pretty tricky. [H3] |

**Figure**

**
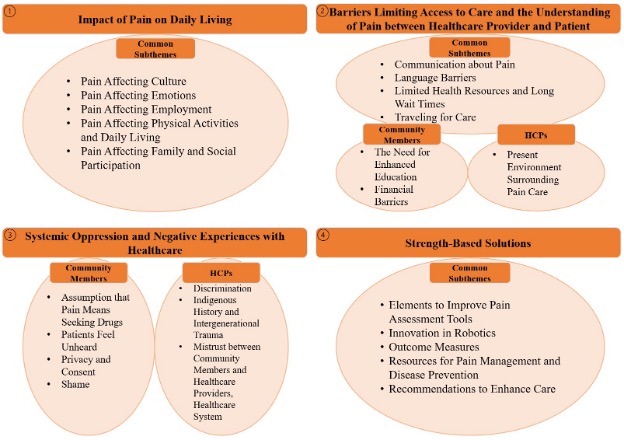
**

**Figure Caption**

**Figure 1 Outline of La Loche Community Member and Healthcare Provider Themes**

The four major themes with their subthemes are outlined. “Common Subthemes” are subthemes which are shared between community members and HCPs. Subthemes which are unique to each group are listed below their respective headings.
